# Crystal structure of di­bromido­(*N*,*N*-di­methyl­formamide-κ*O*){2-(1*H*-indol-3-yl)-*N*-[(quinolin-2-yl-κ*N*)methyl­idene]ethanamine-κ*N*}cadmium

**DOI:** 10.1107/S2056989015000778

**Published:** 2015-01-21

**Authors:** Md. Serajul Haque Faizi, Natalia O. Sharkina, Yuliya M. Davydenko

**Affiliations:** a Department of Chemistry, Indian Institute of Technology Kanpur, Kanpur, UP 208 016, India; b National Taras Shevchenko University, Department of Chemistry, Volodymyrska str. 64, 01601 Kyiv, Ukraine

**Keywords:** crystal structure, Cd^II^ complex with IQME, quinolinyl-containing Schiff base, N—H⋯Br inter­actions

## Abstract

In the mononuclear title complex, [CdBr_2_(C_20_H_17_N_3_)(C_3_H_7_NO)], synthesized from the quinoline-derived Schiff base 2-(1*H*-indol-3-yl)-*N*-(quinolin-2-yl­methyl­ene)ethan­amine (IQME), the coordination geometry around the Cd^2+^ atom is distorted trigonal bipyramidal, the axial positions being occupied by the quinoline N atom [Cd—N = 2.401 (3) Å] and one di­methyl­formamide O-atom donor [Cd—O = 2.399 (2) Å]. The equatorial plane is formed by the imine N atom [Cd—N = 2.293 (3) Å] and two bromides [Cd—Br = 2.5621 (8) and 2.5676 (8) Å], with the deviation of the Cd^II^ atom from the equatorial plane being 0.046 (1) Å. An intra­molecular C—H⋯Br inter­action occurs. In the crystal, N—H⋯Br inter­actions generate [101] chains.

## Related literature   

For applications of quinolinyl-containing Schiff bases, see: Motswainyana *et al.* (2013 ▸); Das *et al.* (2013 ▸); Song *et al.* (2011 ▸); Jursic *et al.* (2002 ▸). The present work is part of an ongoing structural study of Schiff base–metal complexes, see: Faizi & Hussain (2014 ▸); Faizi & Sen (2014 ▸); Faizi *et al.* (2014 ▸); Moroz *et al.* (2012 ▸). For properties of *d* ^10^ metal complexes, see: Henkel & Krebs (2004 ▸); Kimblin *et al.* (2000 ▸); Penkova *et al.* (2010 ▸). For related structures, see: Penkova *et al.* (2009 ▸); Petrusenko *et al.* (1997 ▸).
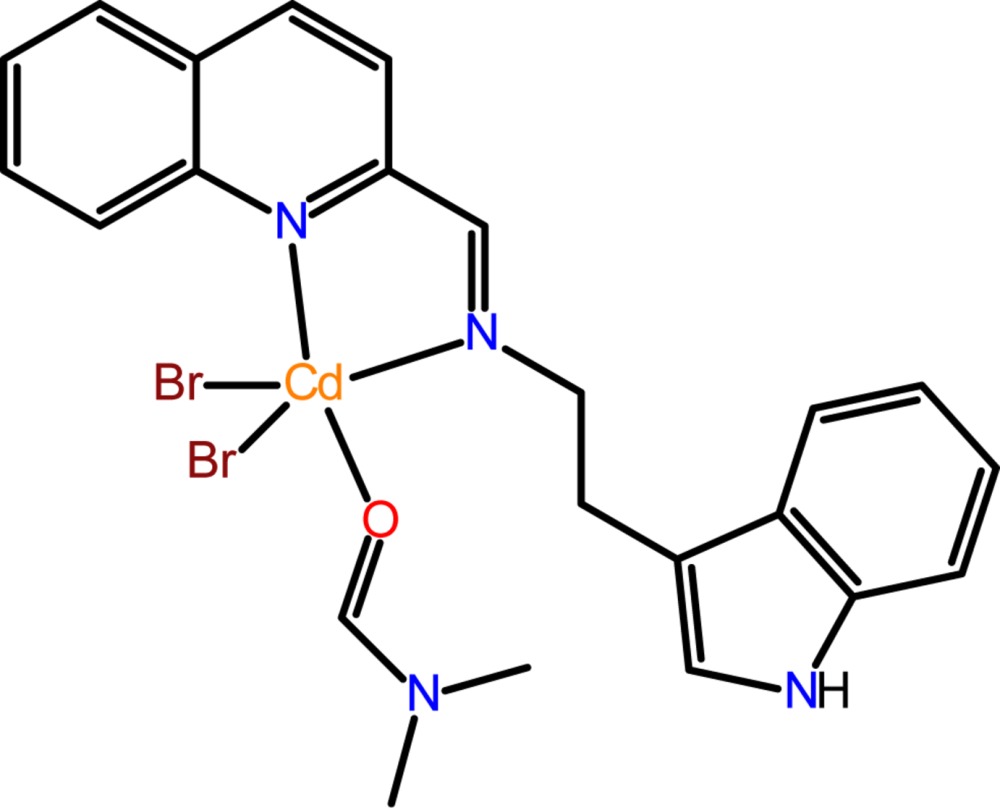


## Experimental   

### Crystal data  

[CdBr_2_(C_20_H_17_N_3_)(C_3_H_7_NO)]
*M* *_r_* = 644.68
Monoclinic, 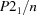

*a* = 14.686 (4) Å
*b* = 8.384 (2) Å
*c* = 20.157 (6) Å
β = 97.785 (5)°
*V* = 2458.9 (12) Å^3^
*Z* = 4
Mo *K*α radiation
μ = 4.16 mm^−1^
*T* = 100 K
0.20 × 0.15 × 0.12 mm

### Data collection  

Bruker SMART APEX CCD diffractometer
Absorption correction: multi-scan (*SADABS*; Bruker, 2003 ▸) *T*_min_ = 0.490, *T*_max_ = 0.635
12172 measured reflections
4308 independent reflections
3120 reflections with *I* > 2σ(*I*)
*R*_int_ = 0.034

### Refinement  

*R*[*F* ^2^ > 2σ(*F* ^2^)] = 0.036
*wR*(*F* ^2^) = 0.092
*S* = 1.02
4308 reflections
286 parameters
90 restraints
H atoms treated by a mixture of independent and constrained refinement
Δρ_max_ = 0.74 e Å^−3^
Δρ_min_ = −0.47 e Å^−3^

### 

Data collection: *SMART* (Bruker, 2003 ▸); cell refinement: *SAINT* (Bruker, 2003 ▸); data reduction: *SAINT*; program(s) used to solve structure: *SIR97* (Altomare *et al.*, 1999 ▸); program(s) used to refine structure: *SHELXL97* (Sheldrick, 2015 ▸); molecular graphics: *DIAMOND* (Brandenburg & Putz, 2001 ▸); software used to prepare material for publication: *SHELXL97*.

## Supplementary Material

Crystal structure: contains datablock(s) global, I. DOI: 10.1107/S2056989015000778/gg2145sup1.cif

Structure factors: contains datablock(s) I. DOI: 10.1107/S2056989015000778/gg2145Isup2.hkl

**Fig. 1.**
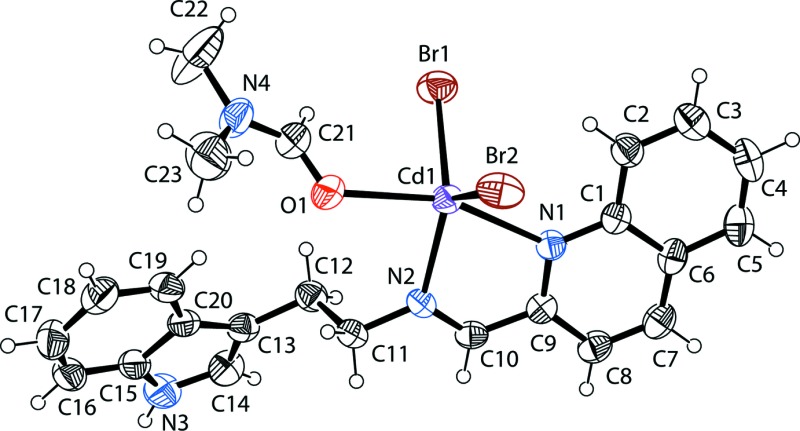
The molecular conformation and atom-numbering scheme for the title compound, with non-H atoms drawn as 40% probability displacement ellipsoids.

**Fig. 2.**
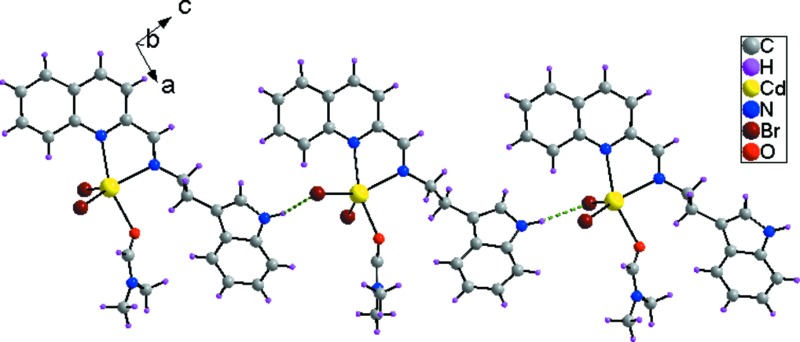
The one-dimensional hydrogen-bonded chain structure in the title compound extending along b, with hydrogen bonds shown as dashed lines.

. DOI: 10.1107/S2056989015000778/gg2145fig1.tif
The mol­ecular conformation and atom-numbering scheme for the title compound, with non-H atoms drawn as 40% probability displacement ellipsoids.

b . DOI: 10.1107/S2056989015000778/gg2145fig2.tif
The one-dimensional hydrogen-bonded chain structure in the title compound extending along *b*, with hydrogen bonds shown as dashed lines.

CCDC reference: 1043591

Additional supporting information:  crystallographic information; 3D view; checkCIF report

## Figures and Tables

**Table 1:** Hydrogen-bond geometry (, )

*D*H*A*	*D*H	H*A*	*D* *A*	*D*H*A*
N3H3*N*3Br2^i^	0.94(3)	2.65(3)	3.504(4)	153(3)
C21H21Br1	0.93	2.83	3.527(5)	132

Symmetry code: (i) 
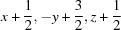
.

## supplementary crystallographic information

### S1. Comment

Quinolyl derivatives of Schiff bases are important building blocks for many important compounds widely used in biological applications such as antioxidative, anticancer, fluorescent probe agents in industry, in coordination chemistry and in catalysis (Motswainyana *et al.* (2013); Das *et al.* (2013); Song *et al.* (2011); Jursic *et al.*(2002). Complexes of d^10^ metal ions such as Zn(II) and Cd(II) are of interest because of their fluorescent properties and involvement in many biological processes (Kimblin *et al.*, 2000; Henkel & Krebs, 2004; Penkova *et al.*, 2010). The synthesis of a complex of cadmium (II) using the quinoline aldehyde derivative of the Schiff base 2-(1*H*-indole-3-yl)-*N*-(quinolin-2-ylmethylene)ethanamine (IQME) has not previously been reported. The present work is part of an ongoing structural study of Schiff-base metal complexes (Faizi & Hussain, 2014; Faizi & Sen, 2014; Faizi *et al.* 2014; Moroz *et al.*, 2012) and we report herein a newly synthesized structure of Cd^II^ complex with IQME, [Cd(Br)_2_(C_20_H_17_N_3_)(C_3_H_7_NO)].

In the structure of title compound, the geometry around the Cd centre can be described as a distorted trigonal bipyramidal, in which the Cd atom is surrounded by one bidentate ligand, two bromines and one DMF molecule coordinated via oxygen (Fig. 1). The axial positions are occupied by N_quinolyl_ of the chelate and O from the DMF molecule [O(1)-Cd(1)-N(1) 160.33 (17) Å], while the equatorial plane is formed by the N_imine_ atom and two bromides, with the deviation of the Cd centre from the equatorial plane of 0.040 Å. The axial plane and the equatorial plane make a dihedral angle of 89.42 Å. The Cd-N_imine_ bond (2.290 (4) Å) is significantly shorter than the Cd1-N_quinolyl_ bond (2.401 (4) Å), which can be attributed to the coordination of the DMF molecule. It is noteworthy that the quinoline ring and indole ring are not coplanar, having a dihedral angle of 35.01 Å. The C—N, C═N and C—C bond lenths are normal and close to the values observed in the related structures (Penkova *et al.*, 2009; Petrusenko *et al.*, 1997). Intermolecular N-H···Br and C-H···Br interactions generate an overall layered structure lying parallel to (010) (Fig. 2).

### S2. Experimental

The iminoquinolyl compound 2-(1*H*-indole-3-yl)-*N*-(quinolin-2-ylmethylene)ethanamine (IQME) was prepared by reacting 2-quinolinecarboxaldehyde with a substituted aniline and was obtained in high yields. This compound was characterized by FT—IR, NMR and ESI-Mass spectroscopy. A mixture of IQME (0.10 g, 0.33 mmol), cadmium(II) bromide (0.09 g, 0.33 mmol) and ethanol (5 ml) were stirred vigorously for 1 h, after which the precipitate was filtered off and redissolved in dimethylformamide. Crystals of the title complex suitable for X-ray analysis was obtained within 3 days by slow evaporation of the DMF solvent.

### S3. Refinement

H atoms were placed in calculated positions and treated as riding on their parent atoms with C—H = 0.95 Å, N—H = 0.88 or 0.91 Å. U_iso_(H) = 1.5U_eq_(N) for the amino-H atoms and 1.2U_eq_(C,N) for the others.

### Crystal data

**Table:**

[CdBr_2_(C_20_H_17_N_3_)(C_3_H_7_NO)]	*F*(000) = 1264
*M**_r_* = 644.68	*D*_x_ = 1.741 Mg m^−^^3^
Monoclinic, *P*2_1_/*n*	Mo *K*α radiation, λ = 0.71073 Å
Hall symbol: -P 2yn	Cell parameters from 2146 reflections
*a* = 14.686 (4) Å	θ = 1.8–25.0°
*b* = 8.384 (2) Å	µ = 4.16 mm^−^^1^
*c* = 20.157 (6) Å	*T* = 100 K
β = 97.785 (5)°	Block, yellow
*V* = 2458.9 (12) Å^3^	0.20 × 0.15 × 0.12 mm
*Z* = 4	

### Data collection

**Table:**

Bruker SMART APEX CCD diffractometer	4308 independent reflections
Radiation source: fine-focus sealed tube	3120 reflections with *I* > 2σ(*I*)
Graphite monochromator	*R*_int_ = 0.034
/w–scans	θ_max_ = 25.0°, θ_min_ = 1.6°
Absorption correction: multi-scan (*SADABS*; Bruker, 2003)	*h* = −17→17
*T*_min_ = 0.490, *T*_max_ = 0.635	*k* = −9→9
12172 measured reflections	*l* = −20→23

### Refinement

**Table:**

Refinement on *F*^2^	Primary atom site location: structure-invariant direct methods
Least-squares matrix: full	Secondary atom site location: difference Fourier map
*R*[*F*^2^ > 2σ(*F*^2^)] = 0.036	Hydrogen site location: inferred from neighbouring sites
*wR*(*F*^2^) = 0.092	H atoms treated by a mixture of independent and constrained refinement
*S* = 1.02	*w* = 1/[σ^2^(*F*_o_^2^) + (0.046*P*)^2^] where *P* = (*F*_o_^2^ + 2*F*_c_^2^)/3
4308 reflections	(Δ/σ)_max_ = 0.001
286 parameters	Δρ_max_ = 0.74 e Å^−^^3^
90 restraints	Δρ_min_ = −0.47 e Å^−^^3^

### Special details

**Table:**

Geometry. All esds (except the esd in the dihedral angle between two l.s. planes) are estimated using the full covariance matrix. The cell esds are taken into account individually in the estimation of esds in distances, angles and torsion angles; correlations between esds in cell parameters are only used when they are defined by crystal symmetry. An approximate (isotropic) treatment of cell esds is used for estimating esds involving l.s. planes.
Refinement. Refinement of F^2^ against ALL reflections. The weighted R-factor wR and goodness of fit S are based on F^2^, conventional R-factors R are based on F, with F set to zero for negative F^2^. The threshold expression of F^2^ > 2sigma(F^2^) is used only for calculating R-factors(gt) etc. and is not relevant to the choice of reflections for refinement. R-factors based on F^2^ are statistically about twice as large as those based on F, and R- factors based on ALL data will be even larger.

### Fractional atomic coordinates and isotropic or equivalent isotropic displacement parameters (Å^2^)

**Table:**

	*x*	*y*	*z*	*U*_iso_*/*U*_eq_	
C1	0.5561 (3)	0.7787 (5)	0.6436 (2)	0.0453 (10)	
C2	0.5466 (3)	0.7981 (6)	0.5739 (2)	0.0566 (12)	
H2	0.5985	0.7951	0.5520	0.068*	
C3	0.4627 (4)	0.8211 (6)	0.5382 (3)	0.0677 (14)	
H3	0.4574	0.8341	0.4920	0.081*	
C4	0.3834 (3)	0.8255 (6)	0.5702 (3)	0.0700 (15)	
H4	0.3263	0.8418	0.5450	0.084*	
C5	0.3891 (3)	0.8065 (6)	0.6373 (3)	0.0649 (13)	
H5	0.3359	0.8083	0.6577	0.078*	
C6	0.4762 (3)	0.7837 (5)	0.6770 (2)	0.0502 (11)	
C7	0.4882 (3)	0.7673 (6)	0.7461 (3)	0.0577 (12)	
H7	0.4372	0.7704	0.7689	0.069*	
C8	0.5739 (3)	0.7469 (6)	0.7813 (2)	0.0554 (12)	
H8	0.5820	0.7370	0.8277	0.067*	
C9	0.6492 (3)	0.7413 (5)	0.7455 (2)	0.0439 (10)	
C10	0.7428 (3)	0.7150 (5)	0.7806 (2)	0.0441 (10)	
H10	0.7511	0.7098	0.8272	0.053*	
C11	0.9037 (3)	0.6785 (5)	0.7898 (2)	0.0469 (11)	
H11A	0.9358	0.5919	0.7711	0.056*	
H11B	0.8962	0.6500	0.8354	0.056*	
C12	0.9611 (3)	0.8304 (5)	0.7905 (2)	0.0522 (11)	
H12A	0.9698	0.8576	0.7450	0.063*	
H12B	0.9284	0.9176	0.8084	0.063*	
C13	1.0533 (3)	0.8096 (5)	0.8323 (2)	0.0495 (11)	
C14	1.0778 (4)	0.8648 (6)	0.8953 (2)	0.0636 (13)	
H14	1.0406	0.9277	0.9185	0.076*	
C15	1.1988 (3)	0.7273 (6)	0.8722 (2)	0.0556 (12)	
C16	1.2826 (3)	0.6473 (7)	0.8738 (3)	0.0686 (14)	
H16	1.3262	0.6495	0.9118	0.082*	
C17	1.2980 (4)	0.5663 (7)	0.8180 (3)	0.0753 (15)	
H17	1.3532	0.5119	0.8182	0.090*	
C18	1.2341 (4)	0.5622 (7)	0.7609 (3)	0.0715 (15)	
H18	1.2477	0.5079	0.7232	0.086*	
C19	1.1504 (3)	0.6380 (6)	0.7594 (2)	0.0583 (12)	
H19	1.1074	0.6339	0.7211	0.070*	
C20	1.1309 (3)	0.7205 (5)	0.8157 (2)	0.0477 (10)	
C21	0.9869 (3)	0.7038 (7)	0.5975 (3)	0.0697 (15)	
H21	0.9706	0.8096	0.5887	0.084*	
C22	1.1131 (5)	0.7539 (10)	0.5352 (5)	0.150 (4)	
H22A	1.1142	0.7114	0.4911	0.225*	
H22B	1.1747	0.7609	0.5580	0.225*	
H22C	1.0859	0.8583	0.5319	0.225*	
C23	1.0901 (5)	0.4887 (8)	0.5815 (3)	0.099 (2)	
H23A	1.1049	0.4468	0.5399	0.149*	
H23B	1.0420	0.4258	0.5962	0.149*	
H23C	1.1436	0.4849	0.6146	0.149*	
Cd1	0.78346 (2)	0.70754 (4)	0.635528 (15)	0.04782 (13)	
N1	0.6416 (2)	0.7570 (4)	0.67886 (17)	0.0435 (8)	
N2	0.8125 (2)	0.6993 (4)	0.75016 (17)	0.0426 (8)	
N3	1.1651 (3)	0.8157 (6)	0.9203 (2)	0.0696 (12)	
N4	1.0598 (3)	0.6510 (6)	0.5720 (2)	0.0701 (12)	
Br1	0.79839 (4)	0.96756 (6)	0.57108 (3)	0.06928 (18)	
Br2	0.72209 (4)	0.46231 (6)	0.56734 (2)	0.06947 (18)	
O1	0.9397 (2)	0.6298 (5)	0.63075 (17)	0.0739 (10)	
H3N3	1.192 (3)	0.842 (5)	0.9637 (13)	0.071 (15)*	

### Atomic displacement parameters (Å^2^)

**Table:**

	*U*^11^	*U*^22^	*U*^33^	*U*^12^	*U*^13^	*U*^23^
C1	0.045 (2)	0.041 (3)	0.050 (3)	−0.002 (2)	0.0079 (19)	−0.004 (2)
C2	0.051 (3)	0.067 (3)	0.050 (3)	0.002 (3)	0.003 (2)	−0.002 (2)
C3	0.066 (3)	0.073 (4)	0.060 (3)	0.001 (3)	−0.006 (2)	0.000 (3)
C4	0.044 (3)	0.076 (4)	0.083 (4)	0.009 (3)	−0.013 (3)	−0.005 (3)
C5	0.043 (3)	0.070 (4)	0.081 (3)	0.005 (3)	0.008 (2)	−0.011 (3)
C6	0.043 (2)	0.043 (3)	0.066 (3)	0.000 (2)	0.012 (2)	−0.007 (2)
C7	0.053 (3)	0.058 (3)	0.066 (3)	0.001 (2)	0.022 (2)	−0.007 (2)
C8	0.058 (3)	0.061 (3)	0.051 (3)	0.002 (2)	0.020 (2)	−0.003 (2)
C9	0.048 (2)	0.040 (3)	0.045 (3)	0.001 (2)	0.0099 (19)	−0.0047 (19)
C10	0.056 (2)	0.041 (3)	0.037 (2)	0.002 (2)	0.0097 (19)	−0.0008 (19)
C11	0.051 (2)	0.046 (3)	0.042 (2)	0.001 (2)	0.001 (2)	0.004 (2)
C12	0.056 (3)	0.046 (3)	0.054 (3)	0.001 (2)	0.005 (2)	0.003 (2)
C13	0.056 (3)	0.047 (3)	0.044 (3)	−0.010 (2)	0.004 (2)	0.001 (2)
C14	0.070 (3)	0.065 (3)	0.057 (3)	−0.012 (3)	0.011 (2)	−0.014 (3)
C15	0.051 (3)	0.064 (3)	0.050 (3)	−0.014 (2)	−0.003 (2)	0.009 (2)
C16	0.056 (3)	0.075 (4)	0.071 (3)	−0.010 (3)	−0.006 (3)	0.020 (3)
C17	0.051 (3)	0.080 (4)	0.094 (4)	−0.004 (3)	0.006 (3)	0.016 (3)
C18	0.067 (3)	0.081 (4)	0.071 (3)	−0.003 (3)	0.025 (3)	0.001 (3)
C19	0.064 (3)	0.068 (3)	0.042 (3)	−0.003 (3)	0.004 (2)	0.004 (2)
C20	0.054 (2)	0.049 (3)	0.040 (2)	−0.009 (2)	0.0054 (19)	0.005 (2)
C21	0.057 (3)	0.090 (4)	0.064 (3)	0.002 (3)	0.017 (3)	0.007 (3)
C22	0.126 (7)	0.142 (7)	0.208 (10)	−0.025 (5)	0.113 (7)	0.009 (6)
C23	0.094 (5)	0.116 (5)	0.089 (4)	0.031 (4)	0.020 (4)	−0.009 (4)
Cd1	0.0476 (2)	0.0570 (2)	0.0402 (2)	0.00472 (16)	0.01071 (14)	0.00239 (15)
N1	0.0435 (19)	0.048 (2)	0.040 (2)	0.0015 (17)	0.0078 (15)	−0.0050 (16)
N2	0.0440 (19)	0.042 (2)	0.0394 (19)	0.0019 (17)	−0.0016 (15)	0.0021 (16)
N3	0.072 (3)	0.089 (4)	0.045 (3)	−0.016 (2)	−0.005 (2)	−0.009 (2)
N4	0.053 (2)	0.092 (3)	0.070 (3)	0.001 (2)	0.025 (2)	−0.005 (2)
Br1	0.0819 (4)	0.0595 (3)	0.0678 (4)	0.0028 (3)	0.0150 (3)	0.0150 (3)
Br2	0.1018 (4)	0.0537 (3)	0.0519 (3)	0.0022 (3)	0.0066 (3)	−0.0024 (2)
O1	0.0531 (19)	0.101 (3)	0.072 (2)	0.0162 (19)	0.0258 (17)	0.026 (2)

### Geometric parameters (Å, º)

**Table:**

C1—N1	1.369 (5)	C14—H14	0.9300
C1—C2	1.401 (6)	C15—N3	1.366 (6)
C1—C6	1.431 (6)	C15—C16	1.397 (7)
C2—C3	1.355 (6)	C15—C20	1.409 (6)
C2—H2	0.9300	C16—C17	1.360 (8)
C3—C4	1.407 (7)	C16—H16	0.9300
C3—H3	0.9300	C17—C18	1.383 (7)
C4—C5	1.352 (7)	C17—H17	0.9300
C4—H4	0.9300	C18—C19	1.381 (7)
C5—C6	1.427 (6)	C18—H18	0.9300
C5—H5	0.9300	C19—C20	1.392 (6)
C6—C7	1.387 (7)	C19—H19	0.9300
C7—C8	1.370 (6)	C21—O1	1.201 (5)
C7—H7	0.9300	C21—N4	1.324 (6)
C8—C9	1.400 (6)	C21—H21	0.9300
C8—H8	0.9300	C22—N4	1.437 (8)
C9—N1	1.338 (5)	C22—H22A	0.9600
C9—C10	1.476 (6)	C22—H22B	0.9600
C10—N2	1.270 (5)	C22—H22C	0.9600
C10—H10	0.9300	C23—N4	1.437 (7)
C11—N2	1.474 (5)	C23—H23A	0.9600
C11—C12	1.526 (6)	C23—H23B	0.9600
C11—H11A	0.9700	C23—H23C	0.9600
C11—H11B	0.9700	Cd1—N2	2.293 (3)
C12—C13	1.505 (6)	Cd1—O1	2.399 (3)
C12—H12A	0.9700	Cd1—N1	2.401 (3)
C12—H12B	0.9700	Cd1—Br1	2.5621 (8)
C13—C14	1.353 (6)	Cd1—Br2	2.5676 (8)
C13—C20	1.439 (6)	N3—H3N3	0.937 (19)
C14—N3	1.376 (7)		
			
N1—C1—C2	119.7 (4)	C17—C16—H16	121.1
N1—C1—C6	120.8 (4)	C15—C16—H16	121.1
C2—C1—C6	119.5 (4)	C16—C17—C18	122.0 (5)
C3—C2—C1	120.6 (5)	C16—C17—H17	119.0
C3—C2—H2	119.7	C18—C17—H17	119.0
C1—C2—H2	119.7	C19—C18—C17	120.6 (5)
C2—C3—C4	120.6 (5)	C19—C18—H18	119.7
C2—C3—H3	119.7	C17—C18—H18	119.7
C4—C3—H3	119.7	C18—C19—C20	119.5 (5)
C5—C4—C3	120.9 (5)	C18—C19—H19	120.3
C5—C4—H4	119.6	C20—C19—H19	120.3
C3—C4—H4	119.6	C19—C20—C15	118.5 (4)
C4—C5—C6	120.4 (5)	C19—C20—C13	134.8 (4)
C4—C5—H5	119.8	C15—C20—C13	106.7 (4)
C6—C5—H5	119.8	O1—C21—N4	127.1 (6)
C7—C6—C5	123.9 (4)	O1—C21—H21	116.5
C7—C6—C1	118.0 (4)	N4—C21—H21	116.5
C5—C6—C1	118.0 (4)	N4—C22—H22A	109.5
C8—C7—C6	120.9 (4)	N4—C22—H22B	109.5
C8—C7—H7	119.5	H22A—C22—H22B	109.5
C6—C7—H7	119.5	N4—C22—H22C	109.5
C7—C8—C9	118.2 (4)	H22A—C22—H22C	109.5
C7—C8—H8	120.9	H22B—C22—H22C	109.5
C9—C8—H8	120.9	N4—C23—H23A	109.5
N1—C9—C8	123.4 (4)	N4—C23—H23B	109.5
N1—C9—C10	116.2 (4)	H23A—C23—H23B	109.5
C8—C9—C10	120.4 (4)	N4—C23—H23C	109.5
N2—C10—C9	122.9 (4)	H23A—C23—H23C	109.5
N2—C10—H10	118.6	H23B—C23—H23C	109.5
C9—C10—H10	118.6	N2—Cd1—O1	89.09 (12)
N2—C11—C12	111.5 (3)	N2—Cd1—N1	71.98 (12)
N2—C11—H11A	109.3	O1—Cd1—N1	160.51 (12)
C12—C11—H11A	109.3	N2—Cd1—Br1	121.29 (9)
N2—C11—H11B	109.3	O1—Cd1—Br1	93.61 (9)
C12—C11—H11B	109.3	N1—Cd1—Br1	100.11 (9)
H11A—C11—H11B	108.0	N2—Cd1—Br2	121.28 (9)
C13—C12—C11	111.2 (3)	O1—Cd1—Br2	91.67 (10)
C13—C12—H12A	109.4	N1—Cd1—Br2	94.25 (8)
C11—C12—H12A	109.4	Br1—Cd1—Br2	117.24 (3)
C13—C12—H12B	109.4	C9—N1—C1	118.7 (4)
C11—C12—H12B	109.4	C9—N1—Cd1	112.9 (3)
H12A—C12—H12B	108.0	C1—N1—Cd1	127.9 (3)
C14—C13—C20	106.1 (4)	C10—N2—C11	118.9 (4)
C14—C13—C12	126.2 (5)	C10—N2—Cd1	115.6 (3)
C20—C13—C12	127.6 (4)	C11—N2—Cd1	125.5 (3)
C13—C14—N3	111.0 (5)	C15—N3—C14	108.2 (4)
C13—C14—H14	124.5	C15—N3—H3N3	130 (3)
N3—C14—H14	124.5	C14—N3—H3N3	122 (3)
N3—C15—C16	130.3 (5)	C21—N4—C23	121.1 (5)
N3—C15—C20	108.1 (4)	C21—N4—C22	121.7 (6)
C16—C15—C20	121.6 (5)	C23—N4—C22	117.2 (5)
C17—C16—C15	117.7 (5)	C21—O1—Cd1	120.7 (3)
			
N1—C1—C2—C3	−179.4 (4)	C8—C9—N1—C1	−0.4 (6)
C6—C1—C2—C3	−0.1 (7)	C10—C9—N1—C1	178.9 (4)
C1—C2—C3—C4	−0.2 (8)	C8—C9—N1—Cd1	−172.7 (3)
C2—C3—C4—C5	−0.2 (8)	C10—C9—N1—Cd1	6.5 (5)
C3—C4—C5—C6	0.9 (8)	C2—C1—N1—C9	179.0 (4)
C4—C5—C6—C7	178.3 (5)	C6—C1—N1—C9	−0.4 (6)
C4—C5—C6—C1	−1.1 (7)	C2—C1—N1—Cd1	−10.0 (6)
N1—C1—C6—C7	0.6 (6)	C6—C1—N1—Cd1	170.7 (3)
C2—C1—C6—C7	−178.7 (4)	N2—Cd1—N1—C9	−5.4 (3)
N1—C1—C6—C5	−179.9 (4)	O1—Cd1—N1—C9	8.8 (6)
C2—C1—C6—C5	0.7 (7)	Br1—Cd1—N1—C9	−125.2 (3)
C5—C6—C7—C8	−179.5 (5)	Br2—Cd1—N1—C9	116.1 (3)
C1—C6—C7—C8	−0.1 (7)	N2—Cd1—N1—C1	−176.9 (4)
C6—C7—C8—C9	−0.6 (7)	O1—Cd1—N1—C1	−162.6 (4)
C7—C8—C9—N1	0.9 (7)	Br1—Cd1—N1—C1	63.3 (3)
C7—C8—C9—C10	−178.3 (4)	Br2—Cd1—N1—C1	−55.3 (3)
N1—C9—C10—N2	−3.7 (6)	C9—C10—N2—C11	178.1 (4)
C8—C9—C10—N2	175.6 (4)	C9—C10—N2—Cd1	−1.6 (5)
N2—C11—C12—C13	178.7 (4)	C12—C11—N2—C10	−105.1 (4)
C11—C12—C13—C14	−102.0 (5)	C12—C11—N2—Cd1	74.5 (4)
C11—C12—C13—C20	74.1 (6)	O1—Cd1—N2—C10	−171.7 (3)
C20—C13—C14—N3	−0.7 (6)	N1—Cd1—N2—C10	3.6 (3)
C12—C13—C14—N3	176.0 (4)	Br1—Cd1—N2—C10	94.6 (3)
N3—C15—C16—C17	179.5 (5)	Br2—Cd1—N2—C10	−80.3 (3)
C20—C15—C16—C17	2.1 (7)	O1—Cd1—N2—C11	8.6 (3)
C15—C16—C17—C18	0.3 (8)	N1—Cd1—N2—C11	−176.1 (3)
C16—C17—C18—C19	−1.7 (8)	Br1—Cd1—N2—C11	−85.0 (3)
C17—C18—C19—C20	0.7 (8)	Br2—Cd1—N2—C11	100.0 (3)
C18—C19—C20—C15	1.7 (7)	C16—C15—N3—C14	−177.8 (5)
C18—C19—C20—C13	−179.3 (5)	C20—C15—N3—C14	−0.2 (5)
N3—C15—C20—C19	179.0 (4)	C13—C14—N3—C15	0.6 (6)
C16—C15—C20—C19	−3.1 (7)	O1—C21—N4—C23	1.1 (9)
N3—C15—C20—C13	−0.2 (5)	O1—C21—N4—C22	−177.3 (7)
C16—C15—C20—C13	177.6 (4)	N4—C21—O1—Cd1	−155.8 (4)
C14—C13—C20—C19	−178.5 (5)	N2—Cd1—O1—C21	−129.2 (4)
C12—C13—C20—C19	4.8 (8)	N1—Cd1—O1—C21	−142.7 (4)
C14—C13—C20—C15	0.6 (5)	Br1—Cd1—O1—C21	−7.9 (4)
C12—C13—C20—C15	−176.1 (4)	Br2—Cd1—O1—C21	109.5 (4)

### Hydrogen-bond geometry (Å, º)

**Table:**

*D*—H···*A*	*D*—H	H···*A*	*D*···*A*	*D*—H···*A*
N3—H3*N*3···Br2^i^	0.94 (3)	2.65 (3)	3.504 (4)	153 (3)
C21—H21···Br1	0.93	2.83	3.527 (5)	132

Symmetry code: (i) *x*+1/2, −*y*+3/2, *z*+1/2.
