# Supplementary figures and images for: Crystal structure of di­bromido­(N,N-di­methyl­formamide-κO){2-(1H-indol-3-yl)-N-[(quinolin-2-yl-κN)methyl­idene]ethanamine-κN}cadmium
Source: Acta Crystallogr E Crystallogr Commun. 2015 Jan 21;71(Pt 2):m31–2. doi: 10.1107/S2056989015000778 (PMC4384581; doi:10.1107/S2056989015000778)

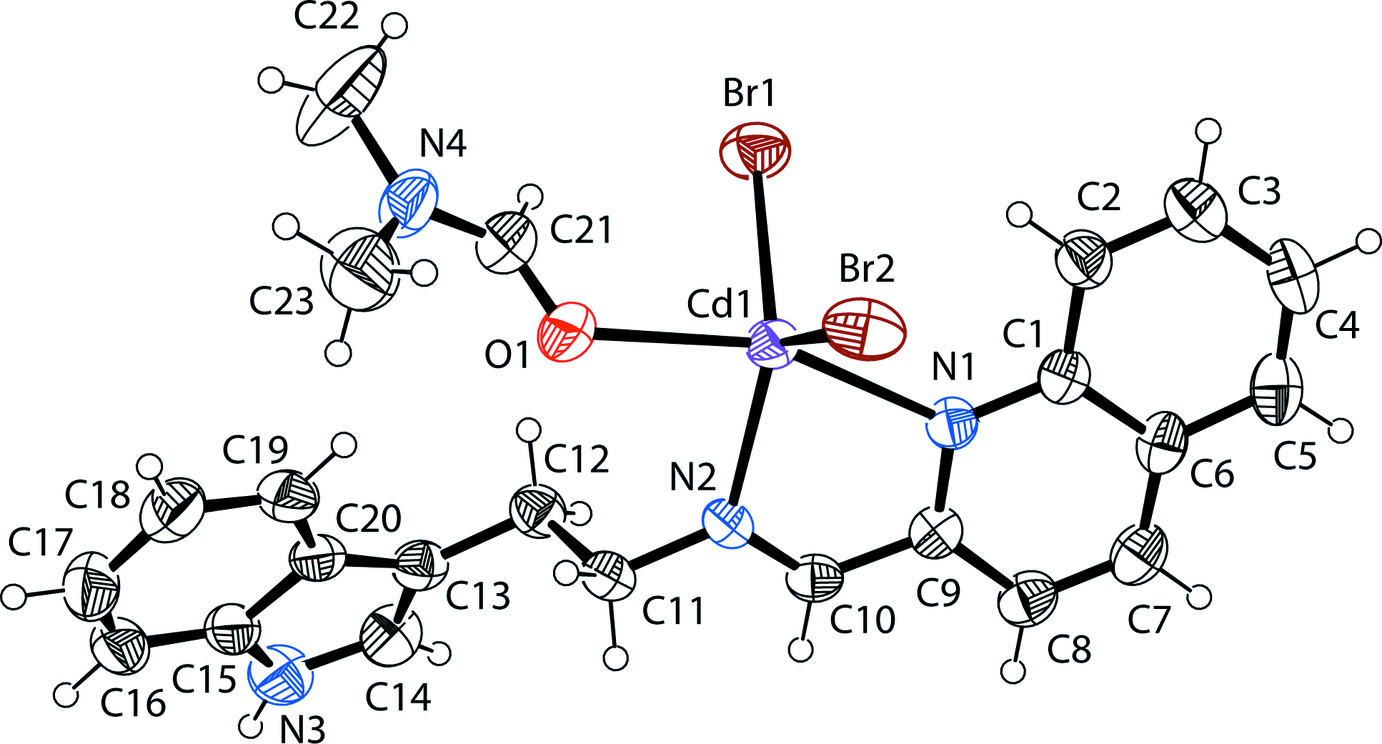

Supplement: Supplementary file 3 [file e-71-00m31-fig1.tif]

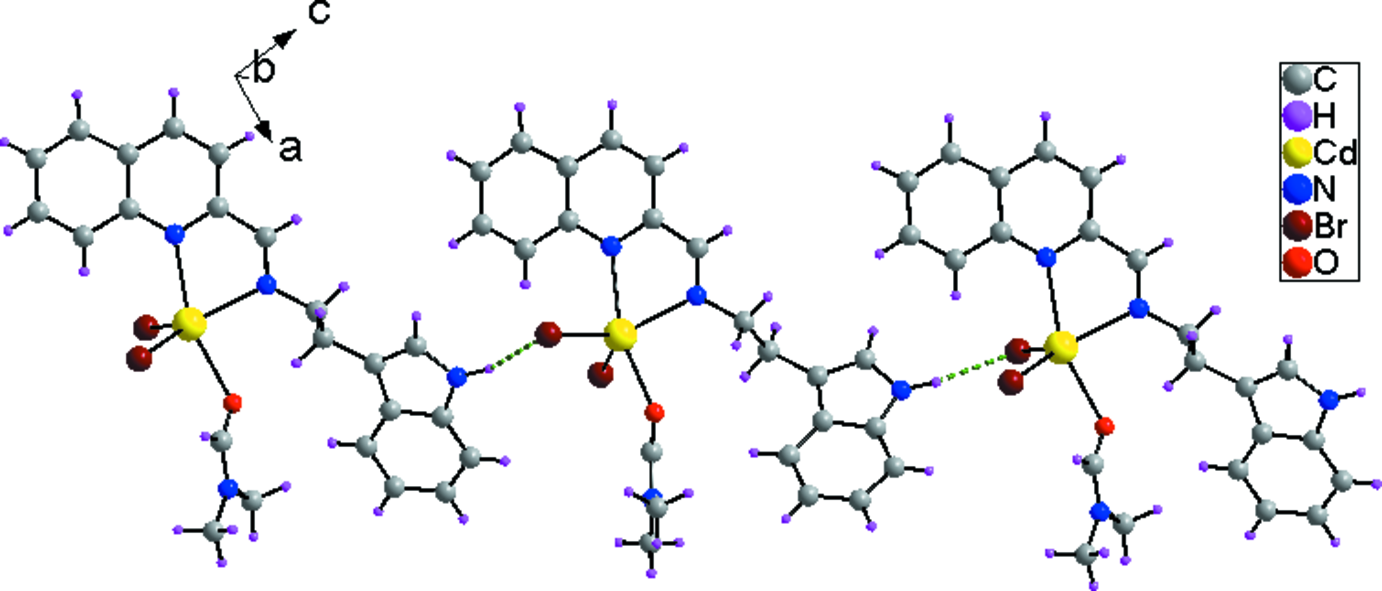

Supplement: Supplementary file 4 [file e-71-00m31-fig2.tif]
